# From anxiety to coping: Understanding psychological distance and coping skills for climate change and COVID-19 in 10–12-year-old children

**DOI:** 10.1371/journal.pone.0317725

**Published:** 2025-02-05

**Authors:** Geertje Schuitema, Anthea Lacchia

**Affiliations:** 1 School of Business, University College Dublin, Dublin, Republic of Ireland; 2 Science Foundation Ireland Research Centre for Applied Geosciences, Dublin, Republic of Ireland; Free University of Bozen-Bolzano: Libera Universita di Bolzano, ITALY

## Abstract

Children worldwide experience climate anxiety, defined as a chronic fear of environmental catastrophe. Following other scholars who sought comparison between the perceived risks and our collective responses with the COVID-19 pandemic, as they can both be considered global crises. Children’s emotional responses, psychological distance and coping strategies to climate change compared and COVID-19 are compared, using a mixed-method longitudinal study among 231 primary school children across the Republic of Ireland. Pre-pandemic and post-lock down data were collected measuring children's emotions through surveys using Likert scales and sketches. Sketching, increasingly recognised as a method for assessing emotional expression, is especially useful when language skills are limited. Results suggest that because climate change was more psychologically distant than COVID-19, it was more difficult for children to effectively use emotional-, meaning-, problem-, and relationship-focused coping strategies to deal with their climate anxiety. One important conclusion of this study is that messages and strategies used to motivate adults to take climate action may increase climate anxiety among young children. Also, relationship-focused coping strategies are underutilised to help children deal with climate anxiety, whilst these were promising coping mechanisms during the COVID-19 pandemic. Therefore, creating positive social support and collective action may help young children to cope with climate anxiety.

## Introduction

Globally children express huge concerns about climate change, which affects their future and triggers concerns about a lack of action to mitigate these climate change. This is expressed through feelings of anxiety, worry, sadness, anger, and powerlessness around the subject of climate change [1–3]. For example, 44% of 10–14-year-olds in Australia were nervous about the future impact of climate change and 27% believed the world may end due to climate change and other global threats [4]. Eighty-seven percent of young Canadians reported to feel that climate change impacts their mental health [2,5] and others found that across 10 different countries, 75% of 16–25 years olds find the future frighting [3]. This paper contributes to this emerging research on climate anxiety among children of the formative age of between 10-and-12-years-old.

Climate anxiety is defined as a chronic fear of environmental doom [6]. Especially children show stronger stress responses to events that are associated with climate change [7], which can causes a range of negative health impacts [8] including their emotional learning, memory, and fear responses [9]. Children are also more at risk of suffering from negative mental health consequences such as post-traumatic stress syndrome, depression, anxiety, phobias, sleep disorders, attachment disorders, which in turn can lead to mental health problems and the inability to regulate emotions later in life [10]. Therefore, the interplay of emotions is core when studying climate anxiety among children [11].

Responses to climate anxiety are believed to alternate between two end points on a continuum [12]. On one end of the continuum, negative climate emotions, such as anger or worry can encourage engagement with climate change and stimulate behavioural change [13–17], if this leads to hope of positive changes [11] or feelings of responsibility [18]. On the other end of the continuum, climate anxiety may lead to in-action, which is described as a type of paralysis when it comes to act on climate change challenges, as they are believed to be unsolvable and intractable [19,20]. As the prospect of acting is likely to be even more difficult for children than for adults, it is arguable even more important for children to find ways to cope with climate anxiety than for adults. Without effective coping mechanisms, children may suffer from long-lasting mental effects. It is important for children to balance their climate anxiety in such a way that it motivates and encourages behaviour change and not lead to a paralysed state of in-action [12]. To achieve this, a good understanding of children's coping strategies and how they can be utilised is needed.

A known type of coping is the creation of psychological distance, a term derived from the Construal Level Theory (CLT). CLT refers to the cognitive distance between oneself and an object or event [21] and assumes that people mentally construe objects that range in how psychologically near or distant they are. Psychologically distant events are usually abstract high-level construals, composed of general decontextualised features. Psychologically close events are presented with concrete low-level construals, which are made up of specific contextual details. Creating psychological distance is a common coping strategy to support thinking about the future. By mentally separating oneself from a situation, flexibility and control over one's thinking and behaviour is created as it creates space to reflect on a decontextualised and abstract event or situation [22]. This suggests that by creating psychological distance between oneself and a source of concern, it is easier to use coping strategies to regulate one's emotions with that concern.

Different dimensions of psychological distance, that is, space, time, social distance, and hypotheticality, affect the mental construal process [21]. *Time* refers to a temporal dimension and indicates that the longer one beliefs an event or situation will take to occur, the larger the psychological distance. *Space* refers to the geographical distance between oneself and situations and events, whereby the further away they are geographically the larger the psychological distance. *Social distance* refers to the perceived psychological proximity or closeness between oneself and others, including those with power: the more power another is perceived to have over and event or situation, the larger the psychological distance that is felt as one perceives less power of these events and situations directly [23]. Finally, *hypotheticality* refers to the perceived likelihood that something will happen in the future whereby psychological distance increases as the likelihood that something will happen reduces. This is related to the time dimension, as the uncertainty around a situation or event will increase further away it is believed to be in the future. 

Studies demonstrate that the larger the psychological distance with respect to climate change, the less concerned people are about climate change, because climate change becomes a more abstract concept [24,25]. However, this has been found from adults and it has been argued that psychological distance with respect to future events has a different effect on children [26,27]. For example, Lee and Atance (27) found that when children thought about a future scenario that involved themselves, psychological distance led to more inaccurate reasoning about the future than when the future scenario involved others. Thus psychological distance with respect to climate change may lead to different coping mechanisms for children compared to adults.

Four types of specific coping strategies can be distinguished [8,28–30]: emotion-focused, problem-focused, meaning-focused and relationship-focused coping strategies. *Emotion-focused coping strategies* rely on de-emphasising and cognitively re-framing the issue and aim to remove or reduce the stressor that leads to negative emotions. As such, this is a strategy to increase psychological distance, and may apply to all four dimensions. Emotional coping strategies can have a short term positive effects but can be counterproductive in the long term [29], especially when there is no solution found to remove the stressor, in this case, if there is no sufficient climate action taken.

*Problem-focused coping strategies* aim to shift the focus away from the issue itself to solutions to the issue that is causing stress, problem-focused coping strategies are generally associated with greater emotional well-being in the long term than emotion-focused coping strategies [29]. However, problem-focused coping strategies have also been associated with stronger negative emotional responses in children [28] possibly because children feel betrayed and abandoned by adults and governments who do not adequately respond to mitigate climate change [3].

*Meaning-focused coping strategies* aim to regulate emotions by activating positive emotions, such as hope and trust in others, whereas *relationship-focused coping strategies* rely on seeking emphatic responses through social support to regulate emotions. For both meaning-focused and relationship-focused coping strategies, promising results were found in that they could be successful coping strategies to mitigate negative emotions and increase levels of well-being, engagement and positive affect [28,31,32].

Recently, the world has been dealing with another global crisis: the COVID-19 pandemic. Pandemics can induce high levels of stress and lead to mental health problems, including depression, anxiety, and stress [33,34]. COVID-19 was also found to lead to large anxiety levels among children [35,36]. Both climate change and COVID-19 are global crises affecting the world in profound and complex ways. Because both crises are global and complex, scholars have sought to draw comparisons between the risks posed by these two crises [37–39] and our collective responses to them [16,40–42]. In this paper, we adopted this approach to assess children's emotional responses, psychological distance and coping strategies to climate change compared to COVID-19. We specifically focus on how the perceived psychological distance between both crises may influence their coping strategies with two specific research objectives.

The first objective is to assess a baseline of both climate anxiety and COVID-19 anxiety levels and which fears and concerns drive these. Emotion appraisal theorists argue that cognitive appraisals about how events in the world relate to one's beliefs, goals, values or knowledge are cause of emotions [43,44]. Understanding the cause of emotions is important to develop coping strategies aimed to minimise negative emotions that are induced by the appraisal of an event like climate change and COVID-19. We focus on the antecedents and consequences of the cause of anxiety, which have been found to be important type of appraisals that have been related anxiety related to climate change [8] and COVID-19 [e.g., [33,45]].

The second objective is to assess how much psychological distance to climate change and COVID-19 is experienced by children and what the implications are to specific coping strategies that they may be using. An increasing amount of literature indicates that coping mechanisms like positive appraisal strategies were effective coping mechanisms that people used during COVID-19 in order to reduce stress and improve mental well-being [46,47]. Our objective is to draw lessons from coping strategies used during the COVID-19 pandemic to help to reduce climate anxiety among children.

## Method

### Study background, procedure, and sample

This study was conducted in 2020, whereby a longitudinal study with a baseline and a follow-up survey were planned to be administered among primary school children in the Republic of Ireland. The study was originally designed as an intervention study aimed to study changes in perceptions of and emotional responses to about climate change among primary school children [48]. However, shortly after the survey was piloted (N = 52) and data collection of the baseline study (N = 179) was finalised in March 2020, all schools were closed as part of COVID-19 regulations. As a result, the planed intervention could not take place. When the schools reopened in September 2020, an amended post-study was conducted, and the follow-up survey included the same questions about climate change as well as questions on COVID-19.

A structured questionnaire with a mixed-methods qualitative and quantitative design was printed as a A5 booklet. The children were guided through the questionnaires in a class setting by 2 members of the research team, which took approximately 20–30 minutes in total. The baseline questionnaire was administered in person by the research team in February and March 2020, thus before the COVID-19 pandemic. The follow-up questionnaire was administered in September and October 2020 through an online call with the classrooms due to COVID-19 restrictions in Ireland. As the procedures and structure were kept identical in both rounds of data collection, and the children and teachers were familiar with the procedures in the follow-up study, there deemed to be no differences in in-person and online guidance in such a way that it affected the data collection.

Recruitment took place through school principals and teachers. A letter of information detailing the study background and methods and an informed consent form were sent to parents before the school visits and the children were given the option to withdraw from taking part at various points throughout the study. No incentives were offered for participation. Full ethical approval was received from the Office of Research Ethics University College Dublin, subsection Human Research Ethics Committee [approval number: HS-20-13-Lacchia] in advance of data collection. Data collection was undertaken following the ethical guidelines, and consent from both the children and their parents or guardians was obtained.

The participants in our sample were in fifth and sixth class, which meant they fell in the age group of between 10–12-years-olds. They were recruited from seven primary schools in Ireland, in four different counties encompassing both rural and city areas. One hundred and seventy-nine participants took part in the baseline questionnaire, and 143 participants took part in the follow-up questionnaire. In total, 75 questionnaires were matched, which means that the baseline and follow-up survey was filled in by the same respondents.

### Measures

To measure how children perceived climate change in the baseline study, the questionnaire began by asking them “Can you sketch what the first thing is that comes to mind when you think of climate change in the box below”. We asked them provide a title with their sketch and to briefly describe what they had drawn and why. The use of drawings or sketches is a method well suited to assess perceptions of various topics [49–52]. The use of drawings is particularly useful in this case, as it requires the expression of emotional expressions [11], and drawings do not rely on formal linguistic capabilities [53], which can be challenging when dealing with 10-12-year-olds. We did invite the participants to write notes and comments in their sketches. The sketches, their titles and descriptions were included in the analyses.

After the sketch, we measured participants' emotional responses to climate change with 5-point Likert-scales, which included a mixture of positive (safe, good, and happy) and negative emotions (upset, angry, and worried). We asked them “When you think about climate change, how does this make you feel?”. The answering scales ranged from 1 (very upset/ good/ angry/ safe/ worried/ happy) to 5 (not at all upset/ good/ angry/ safe/ worried/ happy) and were accompanied by corresponding emoticons to illustrate the meaning of the emotions.

A similar approach and design was used in the follow-up questionnaire. Respondents were first asked to sketch COVID-19. Specifically, they were asked: ‘What’s the first thing you think of when you think of COVID-19? Can you please draw that for us?’ The introduction sentences was added to acknowledge that the children had recently returned to school after they had been closed for six months. They were also asked to add a title and to provide an explanation of the drawing as in the baseline study. Next, the same 5-point Likert-scales were used to measure the six different emotions towards COVID-19 (i.e., upset/ good/ angry/ safe/ worried/ happy).

Next, just as in the baseline questionnaire, they were then asked to sketch climate change and provide a title and explanation. After that, with exactly the same wording and scales as before, they were asked to rate the six emotions again.

### Analyses

Thematic analysis was used to analyse the sketches, the titles of the sketches and the explanations provided alongside them [54,55], which is particularly suited to nuanced, meaning-rich data and is therefore commonly used to analyse this type of data [53]. A coding frame was created to capture important pre-determined featured in the sketches, titles and descriptions. We followed the six steps of thematic analysis that [56] recommend: familiarising with data (1), generating initial codes (2), searching for (sub-)themes (3), reviewing (sub-)themes (4), defining and naming (sub-)themes (5), and assessing the data (6).

The initial coding covered the main theme that was represented in the sketches on climate change (humans, animals, mitigation strategies, local events, or global events) and COVID-19 (humans, animals, virus, mitigation strategies, local events, or global events). Next, coding followed five sub-themes.

The first three sub-themes were used to capture the emotional appraisal of climate change and COVID-19 in terms of antecedents and consequences. The first sub-theme was whether the themes represented antecedents (1), consequences (2), or a mixture of antecedents of consequences (3). The second sub-theme was what type of impact they represented (for climate change: apocalypse (1), death/ illness (2; e.g., animals, humans), environmental (3; e.g., weather, temperature, emissions, litter; natural disasters) or social (4, e.g., climate actions, humans who cause climate change, change of behaviour); for COVID-19: apocalypse (1), death/ illness (2; e.g., animals, humans), environmental (3; e.g., emission, pollution) or social (4, e.g., self-isolation, home-schooling, change of own behaviour). Finally, coding included an impression of the emotions expressed (positive (1), negative (2), neutral/ undefined (3)).

The last three sub-themes were designed to capture psychological distance and the expression of emotions and anxiety. When it comes to specific coping strategies, coding was used to capture how psychological distance rated against the four dimensions of psychological distance: time (1), space (2), social distance (3), and hypotheticality (4). The last sub-theme covered evidence of or lack of emotional (1), meaning (2), problem-focused (3), and (4) relationship-focused coping strategies by holistically assessing the sketches, titles and written comments.

An initial screening of the sketches was conducted by both authors to establish the basis for the coding frame, and both authors defined and agreed on the meaning and definitions of the codes. The coding was conducted by manually by both authors independently. After the first 15 sketches were analysed, authors discussed whether changes to the coding frame and their meaning was needed to ensure consistency. After all sketches were analysed, the authors discussed overlaps and divergences in their coding in order to ensure inter-coder consistency [57].

Four specific steps were taken to ensure inter-coder consistency during the coding process. Firstly, the coding frame was developed by both authors before coding began including thorough discussions about definitions and the interpretation of the themes and sub-themes in the coding framework. After that, each author independently coded ten random sketches based on the framework, followed by a discussion to refine the coding frame and discuss initial divergences in the coding process. Next, all sketches were independently coded by both authors followed by a final thorough discussion about overlaps, divergences and themes.

The quantitative data of the emotional responses to climate change and COVID-19 were analysed with SPSS 26. Mean scores were calculated for each of the six emotions. To test if respondents had different emotions towards climate change (in the baseline and follow-up measurement) compared to COVID-19, an ANOVA Repeated Measures analysis was conducted.

## Results

### Emotional responses to climate change and COVID-19

**Fig 1:**
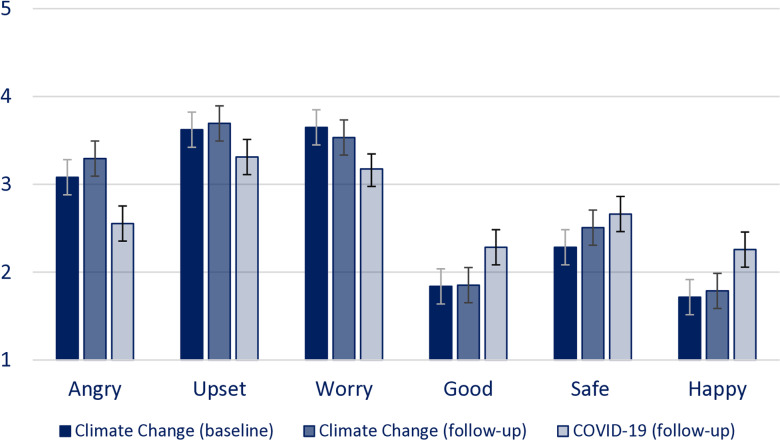
Mean scores on 6 emotions across three conditions. Error bars indicate 95% Confidence Intervals.

To get a baseline understanding of the different emotional responses that participants had towards climate change and COVID-19, a full factorial 6 emotions (feeling upset, worried, angry, good, safe and happy) by 3 conditions (climate change (baseline and follow-up) and COVID) ANOVA Repeated measure was conducted (Table 1 and Fig 1 for mean scores and the statistical data).

**Table 1:** ANOVA Repeated measures testing mean differences between 6 emotions across three conditions (climate change baseline (CC_B), climate change follow-up (CC_F), and COVID-19 follow-up (C19_F)).

	CC_B	CC_F	C19_F
	M (SD)	M (SD)	M (SD)
Angry	3.08 (1.30)	3.29 (1.12)	2.55 (1.11)
Upset	3.62 (1.08)	3.69 (1.11)	3.31 (1.15)
Worry	3.65 (1.22)	3.53 (1.24)	3.18 (1.22)
Good	1.84 (0.91)	1.85 (0.75)	2.28 (0.93)
Safe	2.28 (1.05)	2.51 (1.06)	2.66 (1.16)
Happy	1.72 (1.00)	1.79 (1.02)	2.26 (1.18)

**Multivariate Test**

F (2, 216) = 80.11, p <.001; ηp2 = 0.54

**Univariate Tests**

Emotions (within-subjects): F = (5, 1100) = 89.41, p <.001; ηp2 = .29

Condition (between-subjects): F = (2, 220) = 0.86, p = .425; ηp2 = .01

Emotions*Condition (interaction): F = (10, 1100) = 5.02, p <.001; ηp2 = .05

Note: Analysis only included participants who filled out both baseline and follow-up survey (N = 75).

When comparing the emotional responses towards climate change between the baseline and follow-up participants three negative emotions (angry, upset, worry) were significantly stronger than their positive emotions (good, safe and happy). Within the negative emotions towards climate change, participants felt significantly less angry than that they felt upset and worried. Within the positive emotions towards climate change, they felt safer than they felt good or happy.

For COVID-19, participants were more upset and worried than that they felt good, safe, and happy. There was no significant difference between how angry they felt compared how good, safe, and happy they felt. Similar to climate change, they felt safer than they felt good or happy.

Wilks’ Lambda results indicated that there were significant differences in participants emotional responses to climate change compared to COVID-19 (Table 1). Pairwise comparisons showed that compared to COVID-19, participants overall felt more angry, more upset, more worried, less good, and less happy (Fig 1). There were mixed results with respect to how safe they felt, in compassion to the baseline measure of climate change, participants felt less safe about climate change than about COVID-19, however this difference was not significant when using the follow-up measure for climate change.

Overall, the qualitative data suggest that they felt stronger negative emotions (anger, worry, upset) and less positive emotions (good, happy) when thinking about climate change compared to COVID-19. This indicates that levels of climate anxiety are higher than COVID-19 anxiety levels. However, it is important to note the strongest feelings about COVID-19 were feelings of being worried and upset, which indicates that COVID-19 also was a cause of negative emotional responses among our participants.

### Cognitive appraisal of consequences and antecedents

Thematic analysis confirmed a divide in positive and negative emotions towards climate change and COVID-19. Sometimes, explicit emotions towards climate change and COVID-19 were directly articulated in the sketches. For example, one sketch was titled “Climate change is frustration” (#103, climate change follow-up), but, in line with emotion appraisal theory, most emotions came through cognitive appraisals of consequences and antecedents of both crises.

#### Perceived consequences of climate change.

**Fig 2:**
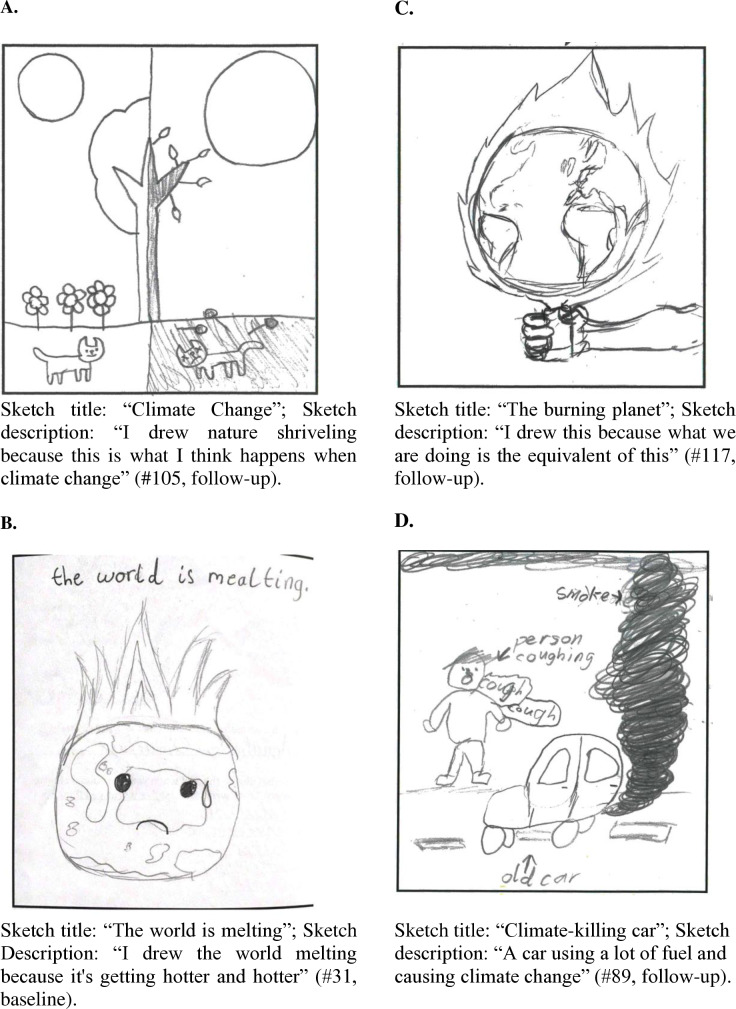
Sketches on perceived consequences (2a and 2b) and perceived antecedents (2c and 2d) of climate change. (A) First illustration of perceived consequences of climate change. (B) Second illustration of perceived consequences of climate change. (C) First illustration of perceived antecedents of climate change. (B) Second illustration of perceived antecedents of climate change.

The perceived consequences of climate change were expressed very clearly in the sketches, and they generally represent very negative situations and events which coincided with negative emotions. About 40% of all climate change sketches represented death and illness, indicating that death and illness was an important sub-theme. The majority of these were sketches of ill or dying animals or a dying Earth, nature, plants, trees, or ecosystem. In a few cases ill or dying humans were represented (Fig 2A). Related is the theme of destruction, catastrophe and apocalypse of the Earth, which was often illustrated by the Earth being on fire, melting, suffocating, going dark, in distress, ill, or dying (Fig 2B). These sketches also represent the perception that climate change has very serious consequences and that the future of the Earth and all that lives on it, is at stake. In addition, many sketches focus on specific consequences of climate change. Themes that were often sketched as consequences of climate change were sea level rises, flooding, wildfires, melting ice caps, flooding, temperature rises, and extreme weather events. These sketches often coincided with suffering of animals, people and nature (Fig 2A). Some of these events were most likely linked to news events, like the wildfires in Australia which were extensively covered in the media during data collection.

In nearly all sketches on climate change expressions of sadness, anger, worry, frustration, powerlessness, and anxiety were observed, for example in the form of tears, sad and angry faces of either the animals, Sun or Earth. Overall, a fear of an existential threat was seen in the vast majority of the climate change sketches which highlights high levels of climate anxiety among our sample.

#### Perceived antecedents of climate change.

Human behaviour was a frequently mentioned antecedent of climate change. There were very generic references to the human cause of climate change (e.g., a hand setting fire to the Earth, see Fig 2C) as well as concrete references to luxury lifestyles, production of goods and electricity. Others indicated that the transportation system, cars and that factories caused smoke (CO_2_) emissions, gasses, fuels (Fig 2D) and therefore damage or hurt the world.

That human behaviour is seen as the cause of climate change is a source of anger, frustration and despair among the participants. A mixture of these emotions was reflected in the expressions of the urgency to change human behaviour to stop climate change (e.g., “I drew this because our world is getting ruined and we don't change now our world will be nothing, #192, baseline), with some very explicit pleas for help (e.g., “please help our planet” #195, baseline) and dire fears and warnings as to what will happen if “we” do not stop climate change (#78, follow-up). Anger and frustration is directed at people who do “bad” things such as littering, polluting, driving cars, but also at “stupid people who don't believe in it” (#195, baseline). Overall, the sketches that referred to human behaviour as antecedent of climate change showed an overarching sense of urgency to act to avoid a large scale crisis as well as anger, frustration and worry about the lack of action that is taken.

In addition, many sketches on climate change referred to events or situation that are important environmental problems, but unrelated to climate change according to the International Panel of Climate Change (IPCC) definition (that reads “a change in the state of the climate that can be identified (e.g., by using statistical tests) by changes in the mean and/or the variability of its properties and that persists for an extended period, typically decades or longer” [58], p544]). For example, there were many sketches of litter, waste and rubbish causing climate change, plastic pollution causing a melting Earth, or palm oil causing deforestation. Also, antecedents could often not be clearly disentangled from the consequences. Climate change itself was often indicated as the antecedent or causal agent of particular impacts or events. For example, melting ice sheets and deforestation were perceived as an entity that is causing climate change and "killing" the Earth (#192, follow-up). Another commonly sketched antecedent was the Sun, which was usually presented as an evil entity, with rays and depicted as causing distress and death in animals, humans, plants and the Earth as a whole. Finally, in some cases, climate change was equated with the weather, e.g., “climate change is the weather” (#116, follow-up, climate change) or presented as a mixture of different associations with climate change such as polar bears, melting ice, rubbish and plastic, and smoke. This implies that children often associated and conflated environmental issues with climate change, and found it sometimes difficult to make sense of the complexity of climate change.

Overall, the sketches presented an overarching sense of urgency to act on the perceived causes of climate change to avoid a large scale crisis. These sketches often clearly expressed emotions like anger, frustration and worry about the lack of action that is taken. For many children climate change was clearly attributed to humans, for others climate change was a complex topic that they could not fully make sense of. However, the perceived antecedents of climate change generally felt outside of children's control.

#### Perceived consequences of COVID-19.

**Fig 3:**
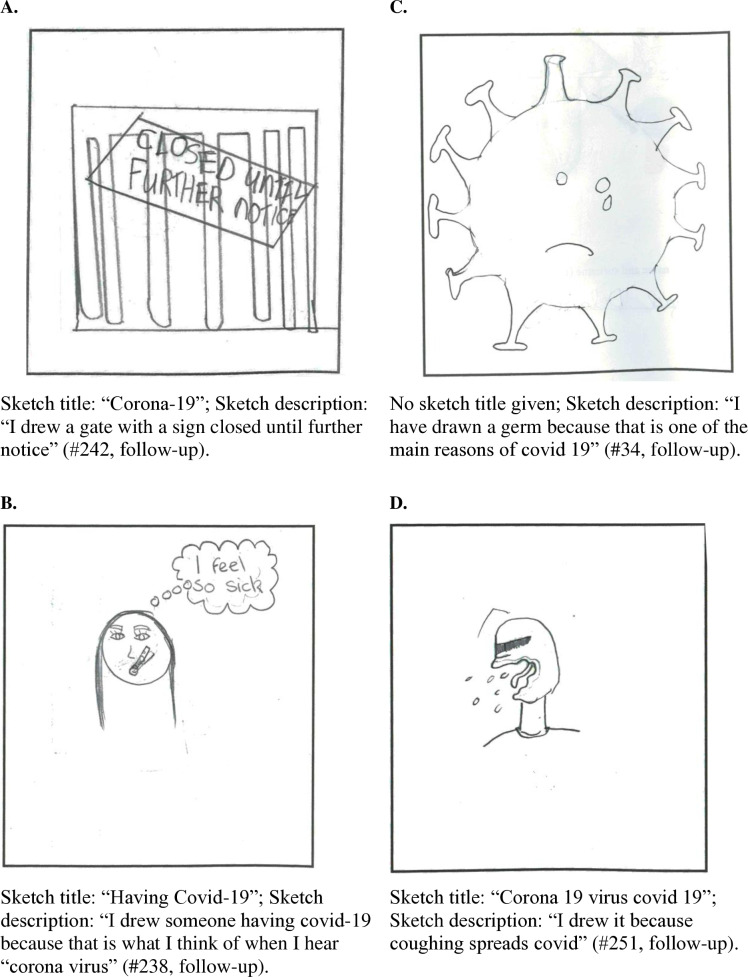
Sketches on perceived consequences (2a and 2b) and perceived antecedents (2c and 2d) of COVID-19. (A) First illustration of perceived consequences of COVID-19. (B) Second illustration of perceived consequences of COVID-19. (C) First illustration of perceived antecedents of COVID-19. (B) Second illustration of perceived antecedents of COVID-19.

A major theme in the sketches was the consequences of COVID-19 on participants' own daily life and local environment during the pandemic. They often sketched being “stuck”, “isolated”, or “locked up” at home. This had consequences for how participants felt. Some sketched indicated feelings of boredom. Sketches on home schooling and not being able to see friends or family reflected feelings of loneliness, because they were missing their friends (“I drew isolation because I could not see my best friend for a while” #57, follow-up). Other consequences for their direct environment were mentioned, such as closed shops, the fact that everyone was wearing face masks, and the widespread use of hand sanitizer, e.g., see Fig 3A).

In contrast to the sketches of climate change, death was not a major theme in the sketches of COVID-19 (For reference, at the end of 2020, the time of data collection, it was estimated that the death rate due to COVID-19 2,230 in Ireland and 1,9 million worldwide [59]). There were very few references to death and funerals. Illness was represented more often, though the consequences of being ill from COVID-19 were usually sketched as pretty mild and short-lived, e.g., sketched as having to lie in bed, having or cough or feeling feverish (see also Fig 3B). Moreover, COVID-19 was presented as sad times for the world because people could not go out, have fun and meet friends.

In conclusion, the majority of consequences of COVID-19 that were drawn reflected the children's personal situation during COVID-19 and how their world changed. This was not a pleasant situation, there was boredom, loneliness and sadness about the situation in the sketches. The national or global scale of the pandemic and all its economic, social and political consequences were not referred to in the sketches.

#### Perceived antecedents of COVID-19.

The antecedents of COVID-19 were clear and straightforward in the sketches, that is a virus or germ was causing an illness: “the emoji represents a sick person with COVID-19 and the germs represent corona” (#13, follow-up; see also Fig 3C). Some referred to a bat that was eaten by a human, which had caused “corona” to develop (#232, follow-up). There were also sketches about how the virus spread among people through coughing (Fig 3D), social contact, and unhygienic behaviours like not wearing masks or not using hand sanitiser. Similarly to climate change, the antecedents of COVID-19 were beyond the control of children, however, there was much more clarity and precision in the presentation of the antecedents of COVID-19 compared to climate change.

#### Psychological distance.

**Fig 4:**
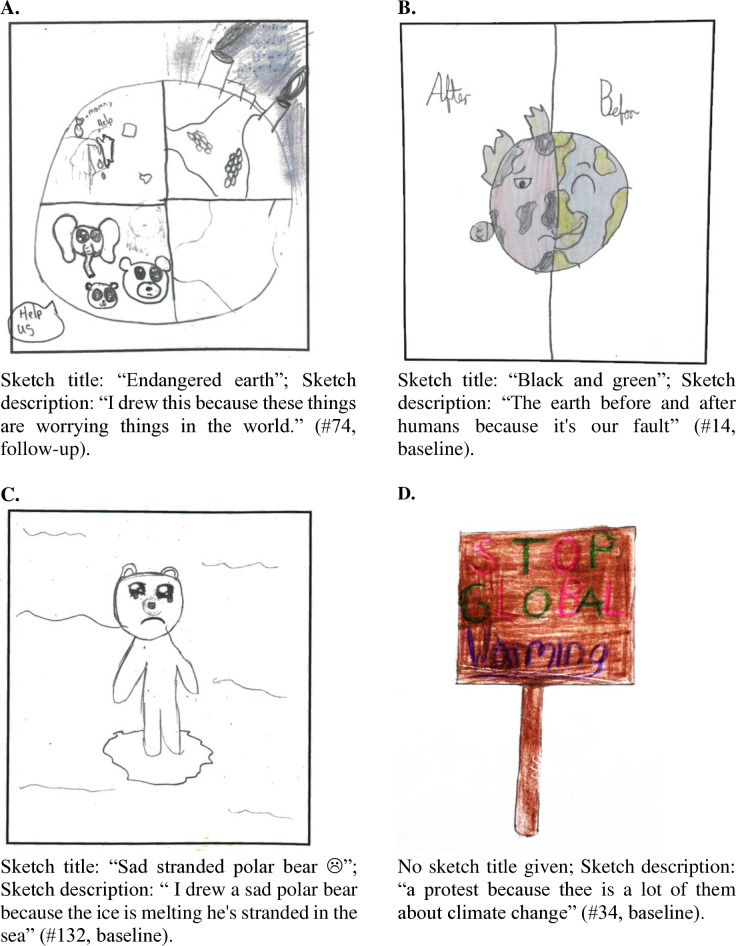
Impression of sketches on four dimensions of psychological distance of climate change. Four dimension of psychological distance refer to (A) place, (B) time, (C) Social distance, and (D) hypotheticality.

**Fig 5:**
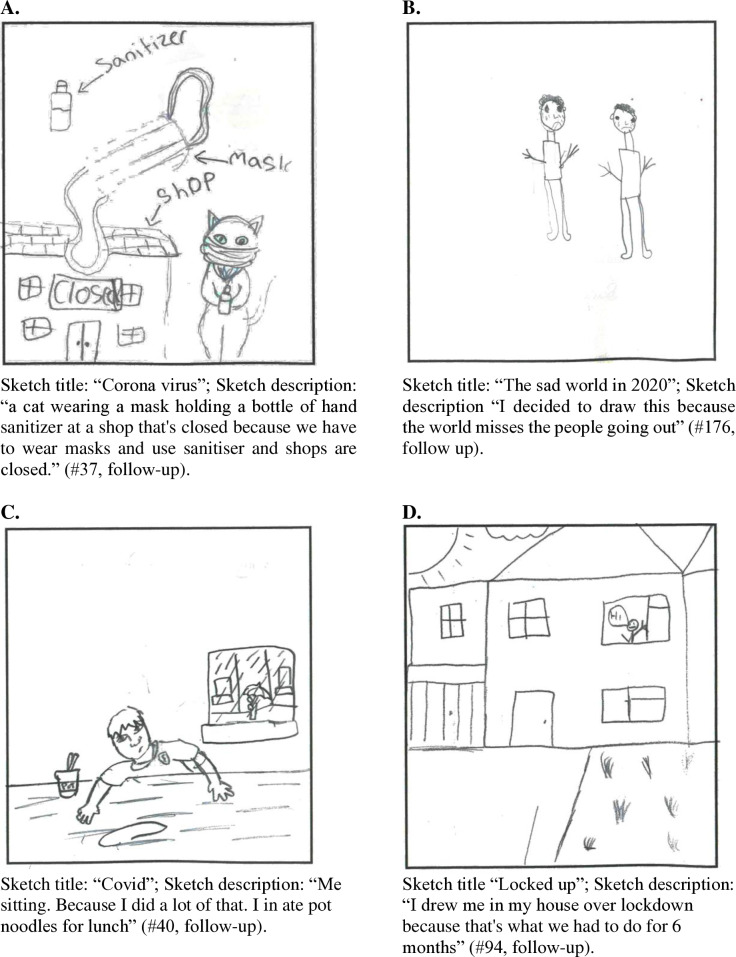
Impression of sketches on four dimensions of psychological distance of COVID-19. Four dimension of psychological distance refer to (A) place, (B) time, (C) Social distance, and (D) hypotheticality.

We assessed four dimensions of psychological distance that were previously distinguished in the context of climate change in our data [24]. *Space* was the first dimension we assessed*,* which refers to physical distance between oneself and the crisis. Climate change was largely sketched as events or situations that occurred far away for Ireland, suggesting a large geographical distance from climate change. For example it was sketched on the poles (melting ice caps), in Australia (wildfires), Africa (snow) or on tropical islands (sea level rise) (see also Fig 4A). Some climate change sketches could potentially be placed at participants' own local environment, which could apply to flooding, changes in weather pattern, and deforestation. However, there were generally no references to Ireland or their personal situations for example in the titles or accompanying descriptions. In contrast, the majority of the sketches about COVID-19 indicated a short geographical distance between participants and the pandemic. The majority of these sketches represented themselves, their daily experiences, and personal observations of the situation (for example see Fig 5A). Thus, we concluded that the geographical psychological distance was larger for climate change than for COVID-19.

The *social distance* dimension referred to whom or what was impacted by the crisis. Climate change was seen to impact the Earth as a whole, including everything and everyone on Earth, such as animals, people, plants, nature. Of all climate change sketches, 50% showed impacts of climate change on animals (e.g., see Fig 4B). Although there was a lot of empathy for the animals impacted by climate change, it does indicate a certain degree of psychological distance, as they are not human and most animals were not native to Ireland (e.g., polar bears, turtles, and orangutans) or a rather broad category like “fish”. In contrast, the *social distance* for COVID-19 sketches was very short, as many sketched focused on how participants were personally impacted. Of the descriptions, 23% referred themselves, for example in terms of “I” (e.g., “… I didn't like the mask”, #9, follow-up), “me” (e.g., “me sitting…”, see Fig 5B), “we” (…we weren't able to see friends… #109, follow-up), or “my” (“I drew my house… #80, follow-up). Thus, we concluded that the social psychological distance was larger for climate change than for COVID-19.

The third dimension was *time*, which referred to how far away an event or situation was on a temporary scale. Many sketches of climate change included a juxtaposition of past or present, good or bad times, or happy and sad periods. In these pictures positive associations are made with the past or present, which represent an Earth unaffected by climate change and is for example sketched or labelled as “good”, “happy”, “healthy”, whilst the future is associated with negative consequences like a dying, suffering and destroyed Earth (Fig 4C). In contrast, the majority of COVID-19 sketches depicted situations that are occurring in the present or near past (Fig 5C), or near future. This suggests that the temporal distance with respect to climate change is larger (both towards its past and its future) than for COVID-19.

Finally, the fourth dimension, *hypotheticality,* referred to the perceived likelihood that something will happen in the future. The level of certainty in the climate change sketches is mainly expressed in terms of “a catastrophe” that will occur “unless action is undertaken”. Whilst there is a lot of certainty that the first part - climate change will lead to a catastrophe – is likely to happen, there is a lot of uncertainty that sufficient action will be taken on time to stop this. This is seen in the urgency in the messages, that proclaim “HELP!” (e.g., “Stop Global Warming”, see Fig 4D), suggesting that participants are very uncertain as to whether this catastrophe will be prevented. If action is too little or too late, most children believed that the catastrophe would occur in their lifetimes. Thus, there was a large level of certainty that climate change would have a negative impact in participants' lifetimes and a large level of uncertainty about the chances of mitigating these impacts. In contrast, the COVID-19 sketches indicated a sense that the crisis would come to an end, which mainly came across in the use of the past tense in the descriptions of the sketches, for example they described that they drew their sketch because “during this time we all had to stay at home” (#22, follow-up), “I could not see my best friend for a while” (#57, follow-up) or “that’s what we had to do for 6 months” (Fig 5D). This suggests that were was a reasonable level of certainty that the COVID-19 pandemic would be resolved in the foreseeable future.

### Coping strategies

#### Coping strategies climate change.

Most sketches on climate change strongly focused on the consequences and antecedents of climate change, which was the main cause of their stress. There were very few indications that children used any coping mechanisms in these sketches. About 10% of the sketches included possible solutions to climate change in their sketches, suggesting a limited form of problem-focused coping strategies. However, the solutions mentioned were typically outside of their control. For example, solutions could be technical, like building wind turbines and the reduction of petrol cars in favour of electric ones, protection of wildlife, nature and biodiversity, reducing transport or stopping pollution and waste. Most solutions that were mentioned were in the hands of others, such as politicians, car drivers, factory owners and people in general. As a result, the sketches that were problem-focused also implied a level of anxiety, urgency and frustration as they often emphasised that solutions had to be implemented in order to prevent a catastrophe.

Very few children demonstrated clear problem-focused coping strategies by referring to solutions they could engage in themselves (an exemption was “a girl planting seeds because lots of people are cutting down trees and plants and that is not good for the environment!” (#184, baseline)). Another form of problem-focused solutions was the use of protests, which was presented as a method to change others, which can be seen as an indirect problem-focused coping strategy. The feeling that they were not listened to in combination with urgency to bring across their message revealed anxiety and frustration (e.g., “What will happen if we don't listen to youth and take action” (#93, baseline)). Inspiration was found in peers: for example references were made to Greta Thunberg who was seen as a source of inspiration: “I drew Greta Thunberg she is inspiring she rebelled and even wrote a speech about it!” (#156, baseline). References to youth movements, protests and Greta Thunberg also suggest a type of relationship-focused coping strategy whereby social support is mainly sought in peers who also experience high levels of climate anxiety.

#### Coping strategies COVID-19.

**Fig 6:**
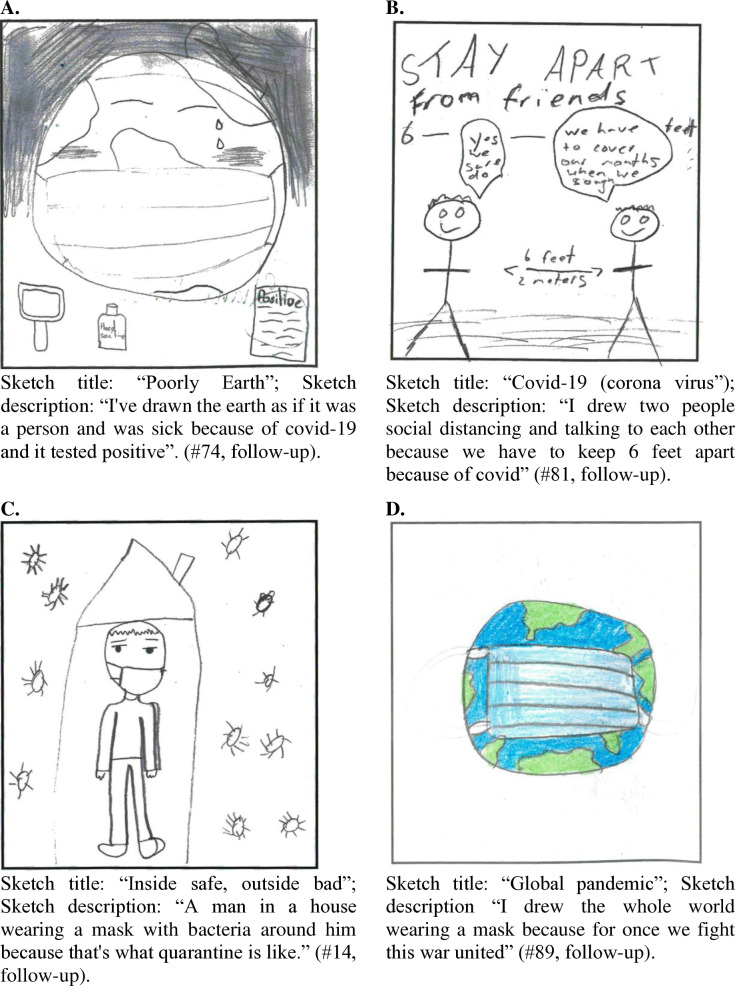
Impression of four coping strategies used to cope with anxiety related to COVID-19. The illustrations refer to (A) meaning-focused coping strategy, (B) problem-focused coping strategies, (C) emotion-focused coping strategies, and (D) relationship-focused coping strategies.

In contrast to the climate change sketches, there were many indications of the use of different coping mechanism in the COVID-19 sketches. Firstly, there were clear signs of *emotion-focused coping strategies* in the sketches. The first sign was the complete absence of the national or global scale of the pandemic and all its economic, social and political consequence, including the hugely polarised debate around the COVID-19 pandemic in the media, politics and the public [60]. If global events were mentioned, it was usually in relatively mild terms like “poorly earth” indicating that there is an illness going around (Fig 6A), which de-emphasised the large amounts of COVID-19 related deaths and suffering that repeatedly in the news. The absence of these topics suggests that children successfully turned their focus away from potentially stressful messages about COVID-19 and instead focused more on their own situation. Their own situation also caused negative emotional responses, such as boredom, loneliness and sadness. However, participants were able to turn that around by focusing on positive outcomes. For example, staying home also made children feel safe (e.g., “I drew me staying at home and keeping my distance to stay safe” #5, follow-up; see also Fig 6B), which is an indication that they used *meaning-focused coping strategies*.

Data also showed clear *problem-oriented coping strategies* by referring to COVID-19 rules and regulations. These sketches typically consisted of drawings of mask-use, hand-sanitising, social distancing, testing, and staying home. The consequences of the rules were not pleasant (e.g., they missed their friends and did not like to wear masks), nevertheless, the sketches showed many smiling faces and compliance with the rules (Fig 6C). Moreover, there was a real sense that the rules and regulations were important solutions to the COVID-19 pandemic, which came through the comments made alongside the sketches, such as “people staying 2 m apart and wearing a face mask which stops COVID-19” (#99, follow-up). This suggests that they believed that following the rules contributed to a solution (e.g., “Because masks is a big help and helps you not get sick and you have to wear it everywhere” #195, follow-up).

Finally, social support was an important feature in the COVID-19 sketches. Social support occurred within households and among friends (e.g., “I drew someone sending their friend a virtual hug in texts, because I do this to all my friends”, #42, follow-up). In addition, there was a feeling of positive collective action to end the pandemic in descriptions like “I drew the whole world wearing a mask because for once we fight this war united” (#89, follow-up) and “we are all in this together” (Fig 6D). The use of the word “war” indicates a level of seriousness to the situation, yet the collective action implies a sense of hope that the crisis would end and that the future would look bright. That they felt they were part of a collective, which gave emotional support, working towards a solution suggests the use of *relationship-oriented coping strategies* on various social dimensions.

### Summary of results

Our data suggested that Irish children between 10 and 12 have severe levels of climate anxiety implying that they chronically fear environmental doom. Whilst children were also seriously worried and upset during the COVID-19 pandemic there was no indication they felt an existential threat and they believed in solutions to the pandemic. Climate change was perceived as a more psychological distant threat than COVID-19 which seems to aggravate their climate anxiety. As COVID-19 was perceived as a psychologically relatively close crisis, children seemed able to use emotional, meaning, problem, and relationship-focussed coping strategies rather effectively to reduce COVID-19 anxiety. To reduce their climate anxiety the data implies that they were far less successful in applying similar coping strategies to reduce climate anxiety.

## Discussion

In line with international trends, our data suggest that Irish children between 10 and 12 have severe levels of climate anxiety implying that they chronically fear environmental doom [1–4], implying that children fear an existential threat in combination with perceived lack of action to mitigate this threat. Our data showed that children perceived climate change as a psychologically distant crisis, which, unlike adults, is related to higher levels of climate anxiety in children [26,27]. The COVID-19 sketches revealed the effective use of a mixture of four coping strategies, that is, emotional-, meaning-, problem-, and relationship-focused coping strategies. However, there was less evidence that children effectively used these four coping strategies successfully to deal with their climate anxiety. We argue that this may be because climate change is a psychologically distant crisis for children, with the caveat that future research is needed confirm such causal relationships.

There were no signs of the systematic use of emotion-focused and meaning-focused coping strategies in the climate change sketches, whilst they have the potential to reduce the fear on an existential threat. The climate change sketches show many similarities with the so-called “disaster narrative” that is often used in climate change messages [61]. Disaster narratives are fear-based narratives that stress the likelihood of devastating outcomes of climate change in a highly emotive message. For example, in these narratives dying animals, especially polar bears, have long been popularised as icons of climate change used to raise awareness and elicit emotional reactions in people by visualising the consequences [62–64]. Such emotive messages have been shown to inspire pro-environmental action and engagement among adults [61,62,64,65]. However, such images of devastation and destruction of animals, ecosystems, and nature can have a counter-productive effect and lead to denial, paralysis, or apathy [66,67], especially among children [20,38]. Without denying the importance and seriousness of climate change, children could be supported to de-emphasise and cognitively re-frame this “disaster narrative”. One could explain that the purpose of these messages is to stimulate those who are responsible for climate change to take action to stimulate emotion-focused coping strategies. This could be a way of providing "constructive" hope [31], which implies a re-focus from problems to positive changes in the past and a trust in others, and stimulating meaning-focused coping strategies.

In line with previous studies, our data suggests that children were effective in using coping strategies to regulate their anxiety about COVID-19 [68]. That is, they focused on practical solutions to the COVID-19 pandemic, such as socially distancing and wearing face masks, which were withing their control. In contrast, the solutions indicated by children for mitigating against climate change were largely beyond their control, such as the pollution caused by others such as cars drivers and industry, and the lack of political action. The main solutions that were within their control concentrated around being heard, in the form of protest and activism. However, this was also a cause of frustration and anxiety, as there was a sense that it wouldn't drive chance fast enough. This suggests that the solution that was within their control, protest, did not appear to be effective in coping with climate anxiety.

Evidence of relationship-focused coping strategies, which rely on seeking empathy and finding social support, were found for both crises. For both crises, social support and empathy was sought and found among friends and peer groups. However, the COVID-19 sketches revealed feelings of positive collective action, implying that children believed that as a collective people in the world would take care of the pandemic. In contrast, in the climate change sketches, a fight between the “good” (climate protesters, those demanding but not in control of change) and the “bad” (the establishment who is not seen to take action to mitigate climate change) can be seen, which creates an in-group and out-group. Previous studies demonstrate that whilst joining or supporting climate protest movements can feel as very empowering and can help to cope with anxiety [15], it can also aggravate anxiety and feelings of frustration and anger if there is a feeling that the action does not lead to sufficient change [3]. As a result, it could be argued that relationship-focused coping strategies that rely on social support from others who experience high anxiety may be a counterproductive coping mechanism to reduce climate anxiety. Instead, it may be more effective to rely on social support from others who experience less climate anxiety and effectively use other coping strategies.

Our data suggests that relationship-focused coping strategies were helpful for children to cope with COVID-19 anxiety. Similarly, focusing on positive social support may help to reduce climate anxiety as well, especially when combined with emotional-, meaning-,and problem-focused coping strategies. For example, research on climate anxiety among children suggests that problem-focused coping strategies should be supported by engaging children in how they can contribute to mitigate climate change, for example by conserving energy, changing their diet and relying less on car transport [69,70]. We argue that combining this with relationship-focused coping strategies whereby these contributions are part of a team or group of people (for example a household, family, class, school, community) may be especially effective. This would create social support and could stress that social networks are contributing to climate change mitigation, which could reduce anxiety (cf.[71]). Similarly, a more collective re-focus from problems to positive changes may help reduce climate anxiety in children as well. Of course, for this to be truly meaningful, it is essential that those who could make a change (e.g., car drivers, industry, policy makers, etc) are seen to be acting too. In line with other studies, we found that in-action of those who could make a change was a major source of climate anxiety and feelings of frustration and betrayal [3,15]. If others appear to be in-active, problem-focused coping strategies may merely enhanced climate anxiety as it does not result in a feeling that real solutions are achieved [cf., [28]].

For children to effectively use relationship-focused coping strategies their social networks are crucial. Parents, teachers, educators, peers and the media all influence the available information and coping mechanism of children [72–74]. Parents, teachers and educators are generally acutely aware of their own climate anxiety as well as their influence on the coping strategies of children [69,74]. They recognised climate anxiety in children and expressed challenges in communicating about this with children, including their own anxiety and concerns about climate change. Our data highlighted that children often associated and conflated environmental issues with climate change. It is important that this is addressed in school curricula, and that teachers and educators can distinguish climate change from other important but often unrelated environmental issues. Overall, there is a clear need for parents, educators and teachers to get support in how to communicate with children about climate change and how to design climate change school curricula [69,70,74]. In addition, clinical professional can contribute to reducing climate anxiety among children and supporting their parents and educators too. Recently, professionals increasingly recognise the importance of addressing climate anxiety as a field that requires independent expertise on how one can support children to adapt to existential threats [75,76]. Our study emphasises the importance of systematically including social dimensions to these practices, in addition to cognitive appraisal and behavioural responses [75].

In this study we focused on climate anxiety among relatively young children (10–12-year-olds). That climate anxiety exists in this age group is well established in the literature. However there is relatively little known about how children in this age group cope with climate anxiety in comparison to adults, Also, as it is a highly emotive topic and it can be difficult, especially for this age group, to articulate in writing their anxiety levels and how they cope with this. To capture their emotions and affective expressions without relying on linguistic capabilities, we asked them to draw their responses [11,53]. Future research could aim to create a better in-depth understanding of the differences between emotional responses of adult and children towards climate change, and how they are intertwined with each other.

Our methodology had limitations too. It is a qualitative research method, which means that the interpretation in terms of the coding framework is conducted by two people. Also, the nature of the data means that we cannot draw conclusions on relationships and causalities. We added some quantitative measurements to validate children's general emotions towards climate change. However, future research would benefit from a methodology to quantitatively capture children's emotional responses to emotive topics to validate the results or our study. We believe that especially the identification of four coping mechanisms forms a good basis for the development of such a methodology.

## Conclusion

Climate anxiety among young children is a growing concern as it negatively impacts their mental well-being. In this paper, we draw lessons from the COVID-19 and evaluate how the coping mechanisms that were used during the COVID-19 pandemic may be applied to reduce climate anxiety. We argue that COVID-19 is perceived as a psychologically closer crisis than climate change, which helps children to effectively use emotional-, meaning-, problem-, and relationship-focused coping strategies to cope with their COVID-19 anxiety. Therefore, reducing the psychological distance with respect to climate change is likely to support the use of these coping strategies. Research suggests that the opposite is true for adults [24,25], and therefore one important conclusion of this study is that it is important to realise that messages and strategies to motivate adults to take climate action may increase climate anxiety among young children. Moreover, we emphasise the potential value of relationship-focused coping strategies for children, which have been largely under-researched in the domain of climate anxiety. Our research suggests that positive social support and collective action was important for children to cope with anxiety during the COVID-19 pandemic. Therefore broad social support, acknowledgement and a feeling of collective action are a promising avenue with which to reduce climate anxiety among children, especially when combined with other coping strategies. We acknowledge that, in order for this strategy to be truly effective, it must include perceived and real social support from policymakers, industry and others who are largely in control of climate mitigation actions.

## Supporting information

S1 File Info sheet and consent form children.
(PDF)

S2 File Info sheet parents and guardians.
(PDF)

S3 File Pre-study survey booklet.
(PDF)

S4 File Post-study survey booklet.
(PDF)

## References

[1] Thompson T. Young people’s climate anxiety revealed in landmark survey. Nature. 2021;597(7878):605–605. doi: 10.1038/d41586-021-02582-8

[2] Galway LP, Field E. Climate emotions and anxiety among young people in Canada: a national survey and call to action. J Clim Chang Health. 2023;9:100204. doi: 10.1016/j.joclim.2023.100204

[3] Hickman C, Marks E, Pihkala P, Clayton S, Lewandowski RE, Mayall EE, et al. Climate anxiety in children and young people and their beliefs about government responses to climate change: a global survey. Lancet Planet Health. 2021;5(12):e863–73. Epub 2021/12/14. doi: 10.1016/S2542-5196(21)00278-3 34895496

[4] Tucci J, Mitchell J, Goddard C. Children’s fears, hopes and heroes: modern childhood in Australia. Australian Childhood Foundation; 2007.

[5] Harper SL, Cunsolo A, Aylward B, Clayton S, Minor K, Cooper M, et al. Estimating climate change and mental health impacts in Canada: a cross-sectional survey protocol. PLoS One. 2023;18(10):e0291303. doi: 10.1371/journal.pone.0291303 37819884 PMC10566728

[6] Clayton S, Manning CM, Krygsman K, Speicer M. Mental health and our changing climate: Impacts, implications, and guidance. Washington, D.C.: American Psychological Association and ecoAmerica; 2017.

[7] Bartlett S. Climate change and urban children: impacts and implications for adaptation in low- and middle-income countries. Environ Urban. 2008;20(2):501–19. doi: 10.1177/0956247808096125

[8] Clayton S. Climate anxiety: psychological responses to climate change. J Anxiety Disord. 2020;74:102263. doi: 10.1016/j.janxdis.2020.102263 32623280

[9] Battaglia S, Di Fazio C, Mazzà M, Tamietto M, Avenanti A. Targeting human glucocorticoid receptors in fear learning: a multiscale integrated approach to study functional connectivity. Int J Mol Sci. 2024;25(2):864. doi: 10.3390/ijms25020864 38255937 PMC10815285

[10] Burke SEL, Sanson AV, Van Hoorn J. The psychological effects of climate change on children. Curr Psychiatry Rep. 2018;20(5):35. doi: 10.1007/s11920-018-0896-9 29637319

[11] Sangervo J, Jylhä KM, Pihkala P. Climate anxiety: Conceptual considerations, and connections with climate hope and action. Glob Environ Change. 2022;76:102569. doi: 10.1016/j.gloenvcha.2022.102569

[12] Hickman C. We need to (find a way to) talk about … Eco-anxiety. J Soc Work Pract. 2020;34(4):411–24. doi: 10.1080/02650533.2020.1844166

[13] Reser JP, Bradley GL, Glendon AI, Ellul MC, Callaghan R. Public risk perceptions, understandings, and responses to climate change and natural disasters in Australia, 2010 and 2011.. Gold Coast, Australia: National Climate Change Adaptation Research Facility; 2012.

[14] Whitmarsh L, Player L, Jiongco A, James M, Williams M, Marks E, et al. Climate anxiety: what predicts it and how is it related to climate action?. J Environ Psychol. 2022;83:101866. doi: 10.1016/j.jenvp.2022.101866

[15] Bright ML, Eames C. From apathy through anxiety to action: emotions as motivators for youth climate strike leaders. Aust J environ educ. 2021;38(1):13–25. doi: 10.1017/aee.2021.22

[16] Verplanken B, Marks E, Dobromir AI. On the nature of eco-anxiety: how constructive or unconstructive is habitual worry about global warming?. J Environ Psychol. 2020;72 101528. doi: 10.1016/j.jenvp.2020.101528

[17] Verplanken B, Roy D. “My worries are rational, climate change is not”: habitual ecological worrying is an adaptive response. PLoS One. 2013;8(9):e74708. doi: 10.1371/journal.pone.0074708 24023958 PMC3762778

[18] Bouman T, Verschoor M, Albers CJ, Böhm G, Fisher SD, Poortinga W, et al. When worry about climate change leads to climate action: How values, worry and personal responsibility relate to various climate actions. Glob Environ Change. 2020;62:102061. doi: 10.1016/j.gloenvcha.2020.102061

[19] Albrecht G. Chronic environmental change: emerging ‘psychoterratic’syndromes. In: Weissbecker I, editor. Climate change and human well-being. New York, NY: Springer; 2011. p. 43–56.

[20] Nagel M. Constructing apathy: how environmentalism and environmental education may be fostering “Learned Hopelessness” in children. Aust J environ educ. 2005;21 71–80. Epub 2015/06/23. doi: 10.1017/s0814062600000963

[21] Liberman N, Trope Y. The psychology of transcending the here and now. Science. 2008;322(5905):1201–5. doi: 10.1126/science.1161958 19023074 PMC2643344

[22] Trope Y, Liberman N. Construal-level theory of psychological distance. Psychol Rev. 2010;117(2):440–63. doi: 10.1037/a0018963 20438233 PMC3152826

[23] Smith PK, Trope Y. You focus on the forest when you’re in charge of the trees: power priming and abstract information processing. J Pers Soc Psychol. 2006;90(4):578–96. doi: 10.1037/0022-3514.90.4.578 16649856

[24] Spence A, Poortinga W, Pidgeon N. The psychological distance of climate change. Risk Anal. 2012;32(6):957–72. doi: 10.1111/j.1539-6924.2011.01695.x 21992607

[25] Leiserowitz AA. American risk perceptions: is climate change dangerous?. Risk Anal. 2005;25(6):1433–42. doi: 10.1111/j.1540-6261.2005.00690.x 16506973

[26] Mazachowsky TR, Koktavy C, Mahy CEV. The effect of psychological distance on young children’s future predictions. Infant Child Dev. 2019;28(4):e2133. doi: 10.1002/icd.2133

[27] Lee WSC, Atance CM. The effect of psychological distance on children’s reasoning about future preferences. PLoS One. 2016;11(10):e0164382. doi: 10.1371/journal.pone.0164382 27741264 PMC5065213

[28] Ojala M. How do children cope with global climate change? coping strategies, engagement, and well-being. J Environ Psychol. 2012;32(3):225–33. doi: 10.1016/j.jenvp.2012.02.004

[29] Lazarus RS, Folkman S. Stress, appraisal, and coping.. New York: Springer Publishing Company; 1984.

[30] O’Brien TB, DeLongis A. The interactional context of problem-, emotion-, and relationship-focused coping: the role of the big five personality factors. J Pers. 1996;64(4):775–813. doi: 10.1111/j.1467-6494.1996.tb00944.x 8956513

[31] Ojala M. Hope and climate change: the importance of hope for environmental engagement among young people. Environ Educ Res. 2012;18(5):625–42. doi: 10.1080/13504622.2011.637157

[32] Cauberghe V, De Jans S, Hudders L, Vanwesenbeeck I. Children’s resilience during Covid-19 confinement. a child’s perspective-Which general and media coping strategies are useful?. J Community Psychol. 2022;50(3):1503–20. doi: 10.1002/jcop.22729 34656070

[33] Mertens G, Gerritsen L, Duijndam S, Salemink E, Engelhard IM. Fear of the coronavirus (COVID-19): predictors in an online study conducted in March 2020. J Anxiety Disord. 2020;74:102258. doi: 10.1016/j.janxdis.2020.102258 32569905 PMC7286280

[34] Mazza C, Ricci E, Biondi S, Colasanti M, Ferracuti S, Napoli C, et al. A nationwide survey of psychological distress among italian people during the COVID-19 pandemic: immediate psychological responses and associated factors. Int J Environ Res Public Health. 2020;17(9):3165. doi: 10.3390/ijerph17093165 32370116 PMC7246819

[35] Meherali S, Punjani N, Louie-Poon S, Abdul Rahim K, Das JK, Salam RA, et al. Mental health of children and adolescents Amidst COVID-19 and past pandemics: a rapid systematic review. Int J Environ Res Public Health. 2021;18(7):3432. doi: 10.3390/ijerph18073432 33810225 PMC8038056

[36] Morrissette M. School closures and social anxiety during the COVID-19 pandemic. J Am Acad Child Adolesc Psychiatry. 2021;60(1):6–7. Epub 2020/09/06. doi: 10.1016/j.jaac.2020.08.436 ; PMCID:PMC7467010 32890669 PMC7467010

[37] Pasini A, Mazzocchi F. Perception and risk of Covid-19 and climate change: investigating analogies in a common framework. Glob Sustain. 2020;3. Epub 2020/12/02. doi: 10.1017/sus.2020.30

[38] Dodds J. The psychology of climate anxiety. BJPsych Bull. 2021;45(4):222–6. Epub 2021/05/19. doi: 10.1192/bjb.2021.18 34006345 PMC8499625

[39] Vercammen A, Oswald T, Lawrance E. Psycho-social factors associated with climate distress, hope and behavioural intentions in young UK residents. PLOS Glob Public Health. 2023;3(8):e0001938. Epub 2023/08/23. doi: 10.1371/journal.pgph.0001938 ; PMCID: PMC10446227 37610987 PMC10446227

[40] Klenert D, Funke F, Mattauch L, O’Callaghan B. Five lessons from COVID-19 for advancing climate change mitigation. Environ Resour Econ (Dordr). 2020;76(4):751–78. doi: 10.1007/s10640-020-00453-w 32836842 PMC7397958

[41] Manzanedo RD, Manning P. COVID-19: lessons for the climate change emergency. Sci Total Environ. 2020;742:140563. doi: 10.1016/j.scitotenv.2020.140563 32619845 PMC7320672

[42] Bouman T, Steg L, Dietz T. Insights from early COVID-19 responses about promoting sustainable action. Nat Sustain. 2020;4(3):194–200. doi: 10.1038/s41893-020-00626-x

[43] Ellsworth PC, Scherer Klaus R.. Appraisal processes in emotion; 2003.

[44] Moors A, Ellsworth PC, Scherer KR, Frijda NH. Appraisal theories of emotion: state of the art and future development. Emot Rev. 2013;5(2):119–24. doi: 10.1177/1754073912468165

[45] Schimmenti A, Billieux J, Starcevic V. The four horsemen of fear: an integrated model of understanding fear experiences during the Covid-19 pandemic. Clin Neuropsychiatry. 2020;17(2):41–5. doi: 10.36131/CN20200202 ; PMCID: PMC8629088 34908966 PMC8629088

[46] Veer IM, Riepenhausen A, Zerban M, Wackerhagen C, Puhlmann LMC, Engen H, et al. Psycho-social factors associated with mental resilience in the Corona lockdown. Transl Psychiatry. 2021;11(1):67. doi: 10.1038/s41398-020-01150-4 33479211 PMC7817958

[47] Kirby LD, Qian W, Adiguzel Z, Afshar Jahanshahi A, Bakracheva M, Orejarena Ballestas MC, et al. Appraisal and coping predict health and well-being during the COVID-19 pandemic: an international approach. Int J Psychol. 2022;57(1):49–62. Epub 2021/06/29. doi: 10.1002/ijop.12770 34189731

[48] Kwasniewska K, Lacchia A, Schuitema G, McElwain J. Tephra bag citizen science project: can volcanic ash help to reduce the amount of CO2 in the air?. Vienna, Austria: EGU General Assembly 2020; 2020.

[49] Chisik Y, editor An image of electricity: towards an understanding of how people perceive electricity. Human-Computer Interaction – INTERACT 2011; 2011. Berlin, Heidelberg: Springer; 2011.

[50] Attari SZ. Perceptions of water use. Proc Natl Acad Sci U S A. 2014;111(14):5129–34. Epub 2014/03/05. doi: 10.1073/pnas.1316402111 ; PMCID: PMC3986180 24591608 PMC3986180

[51] Lacchia A, Schuitema G, McAuliffe F. The human side of geoscientists: comparing geoscientists’ and non-geoscientists’ cognitive and affective responses to geology. Geosci Commun. 2020;3(2):291–302. doi: 10.5194/gc-3-291-2020

[52] Ho ES, Wright FV, Parsons JA. Animated analysis: drawing deeper analytical insights from qualitative data. Int J Qual Methods. 2021;20. doi: 10.1177/1609406921990494

[53] Jensen E. Evaluating children’s conservation biology learning at the zoo. Conserv Biol. 2014;28(4):1004–11. Epub 2014/04/02. doi: 10.1111/cobi.12263 24684607

[54] Marshall C, Rossman G. Designing qualitative research. Sage Publications; 2014.

[55] Boyatzis RE. Transforming qualitative information: thematic analysis and code development. Sage Publications, Inc; 1998.

[56] Braun V, Clarke V. Using thematic analysis in psychology. Qual Res Psychol. 2006;3(2):77–101. doi: 10.1191/1478088706qp063oa

[57] Thomas J, Harden A. Methods for the thematic synthesis of qualitative research in systematic reviews. BMC Med Res Methodol. 2008;8:45. doi: 10.1186/1471-2288-8-45 18616818 PMC2478656

[58] IPCC. Global Warming of 1.5 °C. An IPCC Special Report on the impacts of global warming of 1.5 °C above pre-industrial levels and related global greenhouse gas emission pathways, in the context of strengthening the global response to the threat of climate change, sustainable development, and efforts to eradicate poverty. Cambridge (UK) and New York (USA): Cambridge University Press; 2018.

[59] Mathieu E, Ritchie H, Rodés-Guirao L, Appel C, Giattino C, Hasell J, et al. Coronavirus Pandemic (COVID-19); 2020 [cited 7 Oct 2024]. Available from: Our.World.in.Data.org

[60] Block R Jr, Burnham M, Kahn K, Peng R, Seeman J, Seto C. Perceived risk, political polarization, and the willingness to follow COVID-19 mitigation guidelines. Soc Sci Med. 2022;305:115091. doi: 10.1016/j.socscimed.2022.115091 35690035 PMC9161674

[61] Lowe T, Brown K, Dessai S, de França Doria M, Haynes K, Vincent K. Does tomorrow ever come? Disaster narrative and public perceptions of climate change. Public Underst Sci. 2006;15(4):435–57. doi: 10.1177/0963662506063796

[62] Swim JK, Bloodhart B. Portraying the perils to polar bears: the role of empathic and objective perspective-taking toward animals in climate change communication. Envi Comm. 2014;9(4):446–68. doi: 10.1080/17524032.2014.987304

[63] O’Neill SJ, Hulme M. An iconic approach for representing climate change. Glob Envi Change. 2009;19(4):402–10. doi: 10.1016/j.gloenvcha.2009.07.004

[64] Manzo K. Beyond polar bears? Re‐envisioning climate change. Meteorologi App. 2010;17(2):196–208. doi: 10.1002/met.193

[65] Busch KC. Polar bears or people? exploring ways in which teachers frame climate change in the classroom. Int J Sci Edu Part B. 2015;6(2):137–65. doi: 10.1080/21548455.2015.1027320

[66] Hulme M. Newspaper scare headlines can be counter-productive. Nature. 2007;445(7130):818. doi: 10.1038/445818b 17314953

[67] Feinberg M, Willer R. Apocalypse soon? dire messages reduce belief in global warming by contradicting just-world beliefs. Psychol Sci. 2011;22(1):34–8. Epub 2010/12/15. doi: 10.1177/0956797610391911 21148457

[68] Tambling RR, Tomkunas AJ, Russell BS, Horton AL, Hutchison M. Thematic analysis of parent-child conversations about COVID-19: “Playing It Safe”. J Child Fam Stud. 2021;30(2):325–37. doi: 10.1007/s10826-020-01889-w 33456294 PMC7798006

[69] Baker C, Clayton S, Bragg E. Educating for resilience: parent and teacher perceptions of children’s emotional needs in response to climate change. Envir Edu Res. 2020;27(5):687–705. doi: 10.1080/13504622.2020.1828288

[70] Sanson AV, Burke SEL, Van Hoorn J. Climate change: implications for parents and parenting. Parenting. 2018;18(3):200–17. doi: 10.1080/15295192.2018.1465307

[71] Jones CA, Lucas C. ‘Listen to me!’: young people’s experiences of talking about emotional impacts of climate change. Global Environmental Change. 2023;83:102744. doi: 10.1016/j.gloenvcha.2023.102744

[72] Ojala M. Hope in the face of climate change: associations with environmental engagement and student perceptions of teachers’ emotion communication style and future orientation. J Envir Edu. 2015;46(3):133–48. doi: 10.1080/00958964.2015.1021662

[73] Lawson DF, Stevenson KT, Peterson MN, Carrier SJ, L. Strnad R, Seekamp E. Children can foster climate change concern among their parents. Nat Clim Chang. 2019;9(6):458–62. doi: 10.1038/s41558-019-0463-3

[74] Léger-Goodes T, Malboeuf-Hurtubise C, Hurtubise K, Simons K, Boucher A, Paradis P-O, et al. How children make sense of climate change: A descriptive qualitative study of eco-anxiety in parent-child dyads. PLoS One. 2023;18(4):e0284774. doi: 10.1371/journal.pone.0284774 37079612 PMC10118127

[75] Crandon TJ, Scott JG, Charlson FJ, Thomas HJ. A theoretical model of climate anxiety and coping. Discov Psychol. 2024;4(1). doi: 10.1007/s44202-024-00212-8

[76] Bednarek S. Climate, psychology, and change: Reimagining psychotherapy in an era of global disruption and climate anxiety. North Atlantic Books; 2024.

