# Supplementary material for: From anxiety to coping: Understanding psychological distance and coping skills for climate change and COVID-19 in 10–12-year-old children
Source: PLoS One. 2025 Feb 5;20(2):e0317725. doi: 10.1371/journal.pone.0317725 (PMC11798500; doi:10.1371/journal.pone.0317725)
Supplement: S1 File — (PDF) [file pone.0317725.s001.pdf]

INFORMATION

[e-mail addresses removed for privacy purposes]

Hi, our names are Anthea and Kamilla. We work in University College Dublin and Trinity College Dublin. We would like your help with our research.

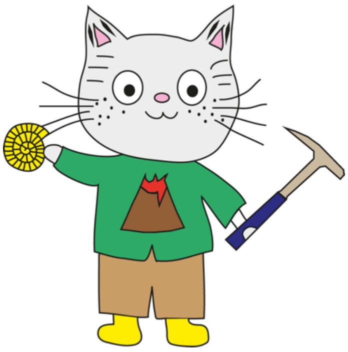

We have asked your parents and they said it is okay for you to talk to us.

Title of the project: Tephra Bag Citizen Science Experiment

What is this about? We are trying to answer this question: can volcanic ash help reduce the amount of CO_2 in the atmosphere? We need your help to find out. We are also interested in your opinions about climate change and the environment.

What is involved? If you agree to take part in this experiment, we will visit you in class twice over the course of three months. We will start by asking you for your opinions on climate change and the environment and then ask you to follow some steps to conduct a scientific experiment. You will be planting seeds in soil and taking various measurements that will help us understand how volcanic ash can help store CO_2 in soils. We will analyse the data you gather and write it up in a scientific report, which you will be able to see.

What is required of you? Nothing outside of class hours. If you agree to take part, you will gather data that will help us with our research.

How will you benefit? You will learn about climate change and about how scientific experiments work.

Are there any risks? None outside of normal, everyday risks.

Do I have to take part? You do not have to take part in this experiment if you don't want to. You won't get into any trouble if you say no. You can change your mind any time about taking part.

Can I ask questions? You can ask questions at any time about any aspect of this project. If you think of a question later, you or your parents can contact us at xxx@xxx.ie (Kamilla) or xxx@xxx.ie (Anthea).

Trinity College Dublin
The University of Dublin

iCRAG
IRISH CENTRE FOR RESEARCH IN APPLIED GEOSCIENCES

UCD DUBLIN

Please answer all of the following (tick the appropriate box):

	**YES**	**NO**
I have read and understood what this project is about and what is required of me	☐	☐
I understand I can decide to stop taking part in this experiment at any point and that I do not have to give a reason.	☐	☐
I agree to take part in this research.	☐	☐

____

Your Signature Printed Name Date

____

Researcher explaining study
Signature Printed Name Date
