# Supplementary material for: From anxiety to coping: Understanding psychological distance and coping skills for climate change and COVID-19 in 10–12-year-old children
Source: PLoS One. 2025 Feb 5;20(2):e0317725. doi: 10.1371/journal.pone.0317725 (PMC11798500; doi:10.1371/journal.pone.0317725)
Supplement: S2 File — (PDF) [file pone.0317725.s002.pdf]

PARENT/GUARDIAN INFORMATION SHEET

[names and contact details removed]

Title of Project: The Tephra Bag Citizen Science Experiment

Who we are: We are xxx (TCD) and xxx (UCD), two postdoctoral researchers from Trinity College Dublin (School of Natural Sciences) and University College Dublin (Business School), respectively. We are both working in iCRAG, the Irish Centre for Research in Applied Geosciences.

What is this research about: This research is investigating perceptions of and possible solutions for climate change.

Why are we doing this research? We are interested in children's perceptions of climate change, including their knowledge, behavior and emotions around climate change. We also want to see if taking part in a scientific experiment about tephra (volcanic ash) has an influence on these perceptions. The experiment involves putting tephra in soil and planting seeds in the soil. The children will track the growth of plants in the soil over three months.

Why has your child been invited to take part? We are looking for primary school children to take part in a citizen science experiment that will help us gather data on climate change. We want to understand children's perception of climate change.

How will the data be used? The data will inform social science research on perceptions of climate change. The information gathered will be treated with privacy and anonymity. No information regarding your child will be stored or revealed in the research. Information will be stored safely with access only available to the research team and examiners and it will all be destroyed after 10 years. The anonymised results from the study may be discussed at conferences or published in a book or a journal.

What will happen if you decide to take part in this study? If you and your child agree to take part in this study, we will visit your child's class twice over a period of three months, and ask your child about their perception of climate change through a questionnaire. The questionnaire, which will take about 30 minutes to complete each time (three months apart) will ask about children's emotions, behaviour, knowledge of climate change. There are no right or wrong answers – we are simply interested in your children's thoughts. All equipment and questionnaire will be provided. The data collected in this study will be stored in a password-protected computer in University College Dublin and archived at the end of the study (31/12/2020).

How will your privacy be protected? We will gather names of children in order to match questionnaires from the same children across our two visits. The names will be stored on an encrypted computer in University College Dublin but the names will be deleted as soon as the second visit it completed and no information regarding your child will be revealed in any report or publication emanating from the research. The information gathered will be treated with privacy and all information, including these consent forms and children's questionnaires, will be stored safely with access only available to the researchers.

What are the benefits in taking part in this research study? Taking part will allow the children to learn about climate change and also take part in a scientific experiment. Taking part involves two classroom visits by the researchers, three months apart. In these three months, the children will learn how to conduct a scientific experiment and gather real scientific data that has the potential to help address climate change. By taking part, they will help gather data that will be analysed by scientists and social scientists.

Photography: The consent form asks you to decide if you are happy for your child to be photographed in class during the researcher's visits. The photos are for publicity on the researchers' and iCRAG's social media channels and on the iCRAG website, and for internal reporting purposes and future funding applications. No names will be associated with the photos. If you do not wish your child to be photographed, this does not in any way preclude your child from taking part in the study.

What are the risks of taking part in this research study? We foresee no risks for your child's participation in the study, beyond those experienced in everyday life.

Can you change your mind at any stage and withdraw from the study? Yes. Your child doesn't have to take part in this study if you/they don't want to and you/they can withdraw from the study at any time, without saying why.

How will you find out what will happen with this project? We will e-mail your school a summary of results of the study as soon as they are available, and they will also be entered into the iCRAG website (www.icrag-centre.org) and a website dedicated to citizen science (Zooniverse).

If you have any questions or concerns, please get in touch with us or with our universities.

Contact details:

Researcher 1 Contact details: ________________________

Researcher 2 Contact details: ________________________

Thank you for taking the time to read this.
