# Supplementary material for: From anxiety to coping: Understanding psychological distance and coping skills for climate change and COVID-19 in 10–12-year-old children
Source: PLoS One. 2025 Feb 5;20(2):e0317725. doi: 10.1371/journal.pone.0317725 (PMC11798500; doi:10.1371/journal.pone.0317725)
Supplement: S3 File — (PDF) [file pone.0317725.s003.pdf]

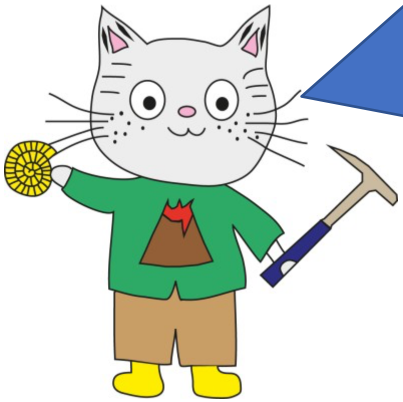

*Hello! We are **Anthea** and Kamilla.*

Will you please help us by answering the questions in this survey? There are no right or wrong answers. We are interested in what you think.

1) Your name <u>and</u> surname (please write clearly):

2) What do you think of when you think about climate change? Please write the first <u>five words you think of</u>:

1._____________________________
2. ____________________________
3. ____________________________
4. ____________________________
5. ____________________________

3) We would like to know what you think off when you think of climate change. Can you sketch what the first thing is that comes to mind when you think of climate change in the box below:

   a. Please give a title to your drawing:

____

b) What did you draw and why? Can you please describe in writing what you have drawn and why?

4) When you think about <u>climate change</u>, how does that make you feel?

Please tick the box that explains how you feel. You can only tick one box. For example, if you feel very upset about climate change, tick the box (like this ☑) below 'very upset'; if you feel a little bit upset, tick the box below 'A little bit upset'.

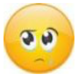	Very upset ☐	Upset ☐	Neither upset or not ☐	A little bit upset ☐	Not at all upset ☐
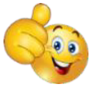	Very good ☐	Good ☐	Neither good or bad ☐	Not so good ☐	Not good at all ☐
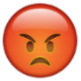	Very angry ☐	Angry ☐	Neither angry or not ☐	A little bit angry ☐	Not at all angry ☐
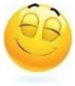	Very safe ☐	Safe ☐	Neither safe or not ☐	A little bit safe ☐	Not at all safe ☐
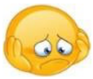	Very worried ☐	Worried ☐	Neither worried or not ☐	A little bit worried ☐	Not at all worried ☐
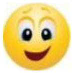	Very happy ☐	Happy ☐	Neither happy or not ☐	A little bit happy ☐	Not at all happy ☐

5) How <u>important</u> is climate change to you?

Please tick the box (like this ☑) below that explains how important it is to you. You can only tick one box.

Very important	Important	Neither important or not	Not important	Not at all important
☐	☐	☐	☐	☐
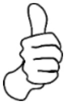	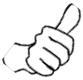	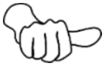	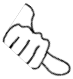	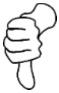

6) How many times have you attended school strikes for climate in the past year at school or outside school?

Write a number; if you have never attended a school strike, write 0.

____

7) How important do you think it is to attend school strikes for climate?

Please tick the box below that explains how important it is to you. You can only tick one box.

Very important	Important	Neither important or not	Not important	Not at all important
☐	☐	☐	☐	☐
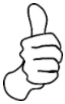	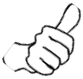	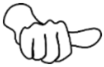	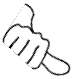	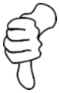

8) Now I am going to ask you to think of a parent, or a guardian. **Just one person**. When you have that person in mind, please answer the following questions:

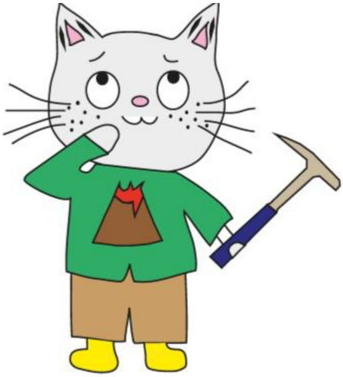

a. Who do you have in mind? Just pick one person.

- ☐ My father
- ☐ My mother
- ☐ Someone else who is my ___________________

b. How do you think <u>this person</u> (your parent or guardian) feels about climate change?

Please tick the box that explains how you think they feel. You can only tick one box.

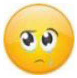	Very upset ☐	Upset ☐	Neither upset or not ☐	A little bit upset ☐	Not at all upset ☐
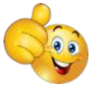	Very good ☐	Good ☐	Neither good or bad ☐	Not so good ☐	Not good at all ☐
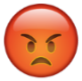	Very angry ☐	Angry ☐	Neither angry or not ☐	A little bit angry ☐	Not at all angry ☐
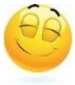	Very safe ☐	Safe ☐	Neither safe or not ☐	A little bit safe ☐	Not at all safe ☐
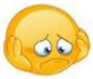	Very worried ☐	Worried ☐	Neither worried or not ☐	A little bit worried ☐	Not at all worried ☐
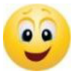	Very happy ☐	Happy ☐	Neither happy or not ☐	A little bit happy ☐	Not at all happy ☐

9) How important do you think <u>this person</u> (your parent or guardian) finds climate change?

Please tick the box that explains your answer. You can only tick one box.

Very important	Important	Neither important or not	Not important	Not at all important
☐	☐	☐	☐	☐
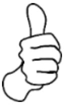	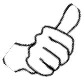	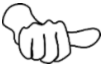	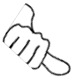	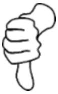

10) Does <u>this person</u> (your parent or guardian) support you in going to a school strike?

Please tick the box that explains your answer. You can only tick one box.

Yes, fully supports me	Somewhat supports me	Neither supports me or not	Not fully	Definitely not
☐	☐	☐	☐	☐
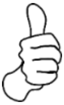	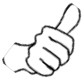	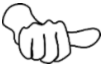	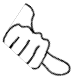	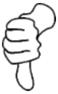

*** PAUSE ***

During introduction and demonstration of experiment. When I ask you, please turn over the page.

11) Now we’ll ask you questions about the Tephra Bag Science experiment. As you heard from the introduction, the Tephra bag project is about changing the carbon cycle. What do you think the carbon cycle looks like? Can you please draw that for us? *Please include as many details as you can remember.*

a. Please give a title to your drawing:

____

b. What did you draw and why? Can you please describe in writing what you have drawn and why?

---

c. What do you think is the aim of this Tephra Bag experiment?

---

d. Why do you think we need an extra pot with no tephra?

---

12) Do you think this will be a fun experiment?

Please tick the box that explains your answer. You can only tick one box.

Very fun	Fun	Neither fun or not	A little bit fun	Not at all fun
☐	☐	☐	☐	☐

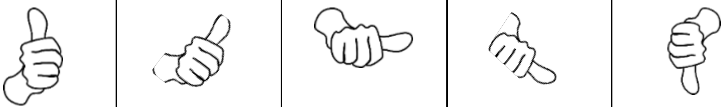

13) Are you excited about this experiment?

Please tick the box that explains your answer. You can only tick one box.

Very excited	Excited	Neither excited or not	A little bit excited	Not at all excited
☐	☐	☐	☐	☐

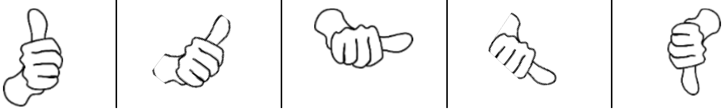

14) Do you think this Tephra Bag Science Experiment is important in addressing climate change?

Please tick the box that explains your answer. You can only tick one box.

Very important	Important	Neither important or not	A little bit important	Not at all excited
☐	☐	☐	☐	☐

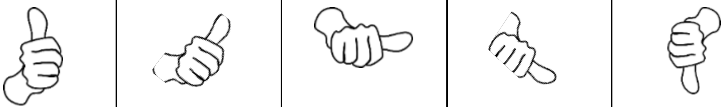

15) Is there anything else you would like to tell us?

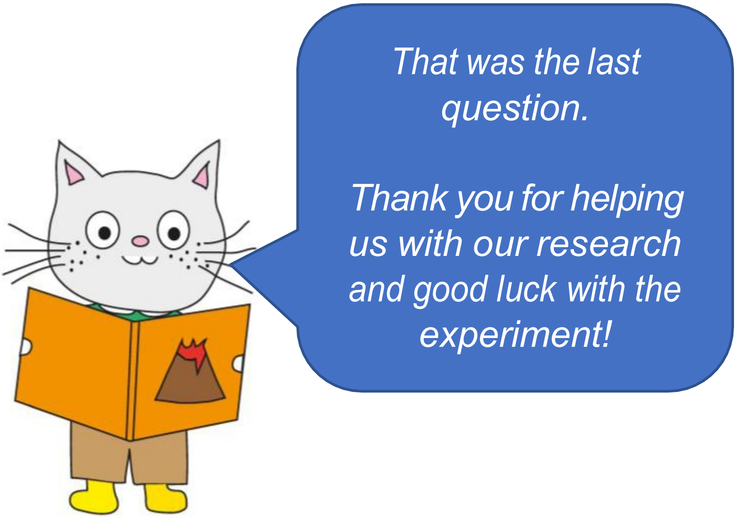

This project is funded by the Irish Centre for Research in Applied Geosciences.

iCRAG IRISH CENTRE FOR RESEARCH IN APPLIED GEOSCIENCES

Trinity College Dublin The University of Dublin

UCD DUBLIN
