# Supplementary material for: From anxiety to coping: Understanding psychological distance and coping skills for climate change and COVID-19 in 10–12-year-old children
Source: PLoS One. 2025 Feb 5;20(2):e0317725. doi: 10.1371/journal.pone.0317725 (PMC11798500; doi:10.1371/journal.pone.0317725)
Supplement: S4 File — (PDF) [file pone.0317725.s004.pdf]

IRISH CENTRE FOR RESEARCH IN APPLIED GEOSCIENCES

Trinity College Dublin
The University of Dublin

UCD DUBLIN

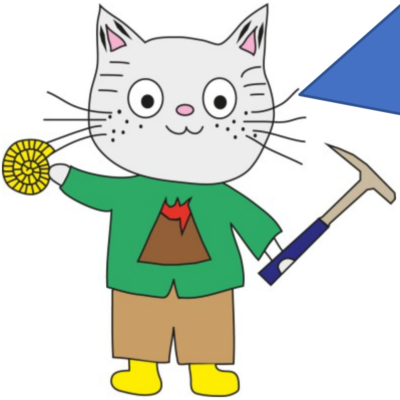

*Hello! We are **Anthea** and Kamilla.*

Will you please help us by answering the questions in this survey? There are no right or wrong answers. We are interested in what you think.

1) Your name <u>and</u> surname (please write clearly):

____

2) Over the past months, the COVID-19 (corona virus) pandemic has affected all our lives. What's the first thing you think of when you think of COVID-19? Can you please draw that for us?

   a. Please give a title to your drawing:

b) What did you draw and why? Can you please describe in writing what you have drawn and why?

3) When you think about the COVID-19 (corona virus) pandemic, how does that make you feel?

Please tick the box that explains how you feel. You can only tick one box. For example, if you feel very upset about COVID-19, tick the box (like this ☑) below 'very upset'; if you feel a little bit upset, tick the box below 'A little bit upset'.

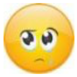	Very upset ☐	Upset ☐	Neither upset or not ☐	A little bit upset ☐	Not at all upset ☐
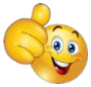	Very good ☐	Good ☐	Neither good or bad ☐	Not so good ☐	Not good at all ☐
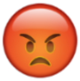	Very angry ☐	Angry ☐	Neither angry or not ☐	A little bit angry ☐	Not at all angry ☐
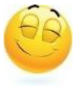	Very safe ☐	Safe ☐	Neither safe or not ☐	A little bit safe ☐	Not at all safe ☐
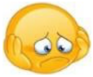	Very worried ☐	Worried ☐	Neither worried or not ☐	A little bit worried ☐	Not at all worried ☐
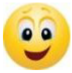	Very happy ☐	Happy ☐	Neither happy or not ☐	A little bit happy ☐	Not at all happy ☐

4) Now, we would like to know what you think of when you think of climate change. Please list the first five words you think of:

1.____________________________

2. ____________________________

3. ____________________________

4. ____________________________

5. ____________________________

5) Please sketch the first thing that comes to mind when you think of climate change in the box below. Please include as many details and labels as you can.

   a. Please give a title to your drawing:

   ____

b) What did you draw and why? Can you please describe in writing what you have drawn and why?

6) When you think about climate change, how does that make you feel?

Please tick the box that explains how you feel. You can only tick one box. For example, if you feel very upset about climate change, tick the box (like this ☑) below 'very upset'; if you feel a little bit upset, tick the box below 'A little bit upset'.

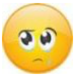	Very upset<br>☐	Upset<br>☐	Neither upset or not<br>☐	A little bit upset<br>☐	Not at all upset<br>☐
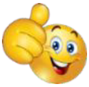	Very good<br>☐	Good<br>☐	Neither good or bad<br>☐	Not so good<br>☐	Not good at all<br>☐
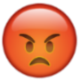	Very angry<br>☐	Angry<br>☐	Neither angry or not<br>☐	A little bit angry<br>☐	Not at all angry<br>☐
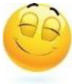	Very safe<br>☐	Safe<br>☐	Neither safe or not<br>☐	A little bit safe<br>☐	Not at all safe<br>☐
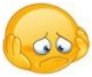	Very worried<br>☐	Worried<br>☐	Neither worried or not<br>☐	A little bit worried<br>☐	Not at all worried<br>☐
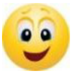	Very happy<br>☐	Happy<br>☐	Neither happy or not<br>☐	A little bit happy<br>☐	Not at all happy<br>☐

7) How <u>important</u> is climate change to you?

Please tick the box (like this ☑) below that explains how important it is to you. You can only tick one box.

Very important	Important	Neither important or not	Not important	Not at all important
☐	☐	☐	☐	☐
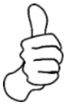	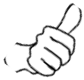	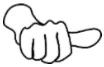	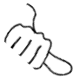	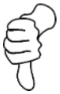

8) Now I am going to ask you to think of <u>a parent, or a guardian</u>. **Just one person**. When you have that person in mind, please answer the following questions:

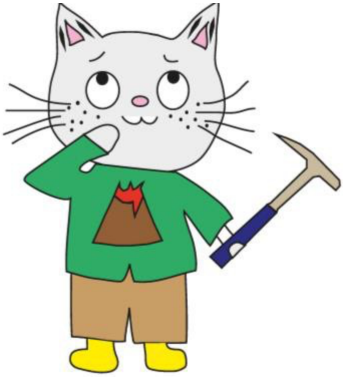

a. Who do you have in mind? Just pick one person.

- ☐ My father
- ☐ My mother
- ☐ Someone else who is my ___________________

9) How important do you think this person (your parent or guardian) finds climate change?

Very important	Important	Neither important or not	Not important	Not at all important
☐	☐	☐	☐	☐
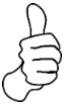	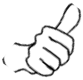	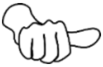	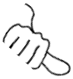	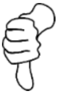

10) Do you think there is a link between the COVID-19 (coronavirus) pandemic and climate change? Please explain.

11) Is there anything else you would like to tell us?

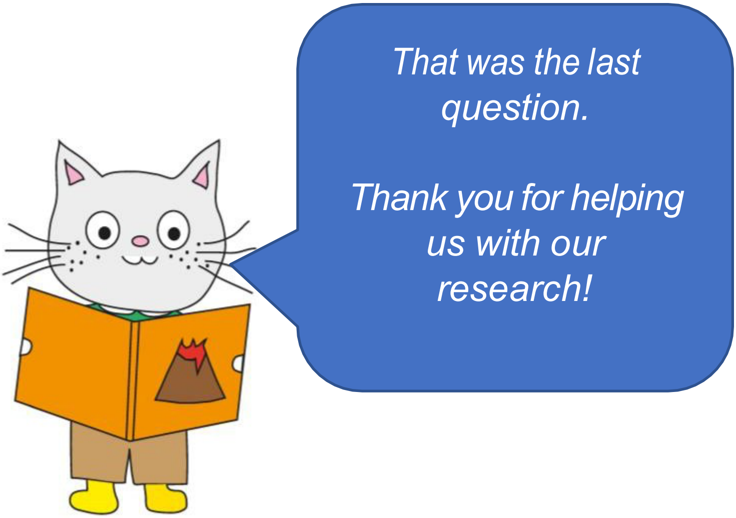

This project is funded by the Irish Centre for Research in Applied Geosciences.

iCRAG IRISH CENTRE FOR RESEARCH IN APPLIED GEOSCIENCES

Trinity College Dublin The University of Dublin

UCD DUBLIN
